# The Landscape of Currently Enrolling Maintenance Trials in Multiple Myeloma

**DOI:** 10.1007/s44228-023-00044-8

**Published:** 2023-05-03

**Authors:** Syed Maaz Tariq, Al-Ola Abdallah, Aaron Goodman, Douglas Sborov, Ghulam Rehman Mohyuddin, Alec Britt

**Affiliations:** 1 Jinnah Sindh Medical University, Karachi, Pakistan; 2 Division of Hematological Malignancies and Cellular Therapeutics, University of Kansas Medical Center, Kansas City, KS USA; 3 Division of Hematology and Oncology, University of California San Diego, La Jolla, CA USA; 4 Division of Hematology and Hematological Malignancies, University of Utah, Salt Lake City, UT USA

**Keywords:** Myeloma, Landscape, Lenalidomide, Maintenance

## Abstract

Maintenance therapies in multiple myeloma improve survival after induction treatment. This study characterizes the strategies for maintenance therapy being employed in currently enrolling clinical trials for patients with multiple myeloma and highlights how high-risk myeloma patients may be assigned to maintenance strategies incongruent with current US guidelines.

Despite dramatic advances in therapeutic options for multiple myeloma (MM), the disease is associated with considerable morbidity and mortality [1]. Current standard first-line therapy for fit and eligible patients with MM involves triplet or quadruplet induction, followed by autologous stem cell transplant (ASCT) and maintenance therapy [2].

Even in the setting of optimal therapy during the first line of treatment, disease relapse is expected [3]. Maintenance therapy post-ASCT delays disease progression and prolongs survival. The current standard for it is lenalidomide, an immunomodulatory drug FDA-approved in 2017 after several large, randomized phase 3 trials demonstrated significant improvement in progression free survival (PFS), and a large meta-analysis confirmed overall survival (OS) benefit [4–6] of its use in the maintenance setting. Although other agents such as daratumumab and ixazomib have been studied as maintenance strategies, no OS benefit has yet been seen [7, 8].

Patients with high-risk cytogenetic features historically have poorer outcomes than patients with standard risk MM. There is mounting interest in the use of combination therapy for maintenance, and doublet maintenance is currently recommended in national USA guidelines for patients with high-risk MM [9, 10]. The recent FORTE trial demonstrated greater PFS with the carfilzomib and lenalidomide combination versus lenalidomide alone, with an improvement in PFS noted in high-risk subset of patients [10]. Furthermore, the role of minimal residual disease (MRD) in maintenance therapy continues to evolve [11] and the use of MRD allows for a platform to evaluate discontinuation of continuous therapy for deep responders.

We sought to assess the current landscape of maintenance therapy in clinical trials for newly diagnosed MM. The objective of this study was to characterize ongoing clinical trials with regards to agent(s) being used, the proportion of randomized studies, and characterization of primary endpoints. We assessed the proportions of currently enrolling randomized studies that were evaluating: OS for a primary endpoint; PFS in direct comparison to lenalidomide, and. MRD in the decision to discontinue treatment. Finally, we assessed whether high-risk MM patients were being enrolled in these studies and the maintenance therapy utilized in these patients.

A comprehensive search was performed on the clinicaltrials.gov, clinicaltrialsregister.eu, and anzctr.org.au databases on March 2, 2022, using the keywords “newly diagnosed multiple myeloma”, “transplant-eligible multiple myeloma”, and “maintenance in multiple myeloma” as detailed in Fig. 1. Studies which were currently recruiting patients, or those which were active but not yet recruiting were included, but not those which had completed enrollment or were terminated. Studies were included if patients were planned to undergo ASCT after induction therapy, followed by maintenance strategy, and if at least one arm received maintenance therapy, with the latter specified in the trial description or interventions. Trials evaluating maintenance therapy after a salvage transplant or maintenance therapy in non-transplant settings, those without a defined maintenance therapy agent(s) and those investigating non-pharmacological interventions were excluded.

**Fig. 1:**
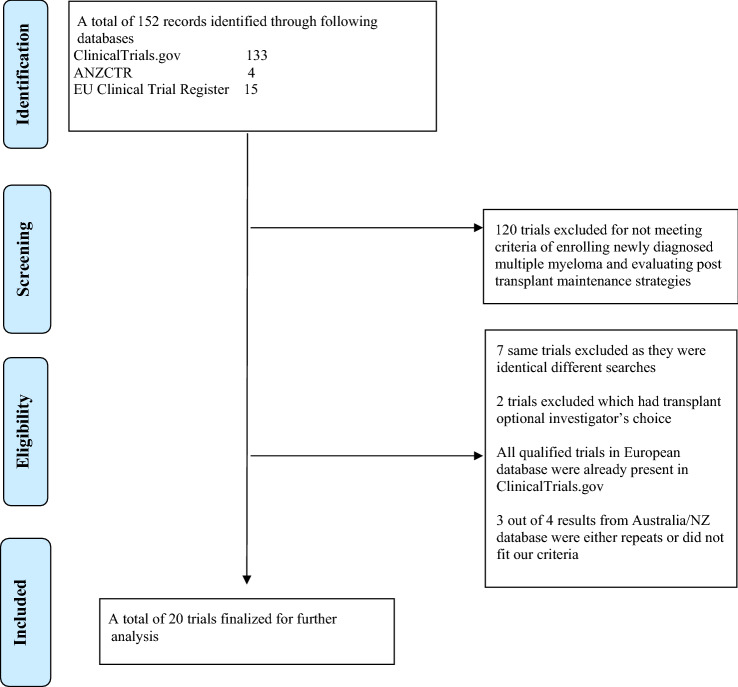
Flow diagram depicting our search strategy and study inclusion

A total of 20 studies were identified and analyzed (Table 1). Of these, 11 (55.0%%) were randomized and nine (45.0%) were non-randomized.

**Table 1:** Study Characteristics

Trial characteristics	*N* (%) (*N = *20)
Randomized	11 (55.0)
Non-randomized	9 (45.0)
Phase II study	12 (60.0)
Phase III study	8 (40.0)
US based enrollment	9 (45.0)
Non-US-based enrollment	11 (55.0)
Studies including high-risk patients	19 (95.0)
Primary end point(s)	
Response based	3 (25.0)
PFS based	6 (30.0)
OS based	2 (10.0)
MRD status	11 (55.0)
Incidence of death based	1 (5.0)
Proportion able to complete 1 year therapy	1 (5.0)
FACT-MM TOI score	1 (5.0)

PFS= progression free survival; OS= overall survival; MRD= Minimal residual disease; FACT-MM TOI= Functional assessment of cancer therapy - multiple myeloma trial outcome index

A time to event outcome (either PFS or OS) was included as a primary endpoint in 8 of the 20 studies (40.0%). Four of the 20 (20.0%) included multiple primary endpoints in addition to PFS or OS. Six studies (30.0%) evaluated PFS and two (10.0%) included OS as primary outcomes. Of the 11 randomized studies, time to event-based was a primary endpoint in 7 (63.6%), with 2 evaluating OS (18.1%) and 5 PFS (45.5%).

MRD negativity was included as a primary endpoint in 11 studies (55.0%) and a secondary endpoint in 7 (35%). Two trials assessed discontinuation of maintenance therapy for those achieving MRD negativity (NCT04071457, NCT05091372). The former trial utilized MRD negativity at 24 months, and the latter utilized sustained MRD criteria defined as two separate MRD-negative evaluations at least 1 year apart.

Of the 9 non-randomized studies, 4 had a primary endpoint of MRD negativity rate (44.4%), two (22.2%) had response-based primary outcomes, and one (11.1%) had a time to event primary outcome.

Table 2 describes maintenance regimens utilized in the included studies. Among the non-randomized studies (*n = *9), a single-drug maintenance strategy was utilized in 5 (55.6%), and combination regimen was utilized in the other 4 (44.4%). Among the randomized trials (*n = *11), the intervention arm comprised of combination therapy in 8 studies (72.7%), and single arm therapy in 3. In the randomized studies (*n = *11), the control arm was lenalidomide in 8, ixazomib in 1, isatuximab in 1 and combination maintenance therapy in 1 (NCT05091372). A total of three randomized studies assessed comparison of lenalidomide and another agent to lenalidomide alone with a primary endpoint powered for PFS (ACTRN12620000291987, NCT05243797, NCT05317416), and 2 randomized studies comparing these had OS as the primary outcome (NCT 04071457, NCT 03941860).

**Table 2:** Maintenance regimens utilized

Nonrandomized studies	Total studies
Single agent maintenance	
Belantamab	1
Daratumumab	1
Iberdomide	2
Lenalidomide	1
Combination maintenance	
Belantamab + lenalidomide	2
Isatuximab + lenalidomide	2

Randomized Studies	Total studies	Control arm maintenance
Single agent maintenance: treatment arm		
Elranatamab	1	Lenalidomide
Lenalidomide	2	Lenalidomide
Combination maintenance: treatment arm		
Belantamab + lenalidomide	1	Belantamab + lenalidomide
Cellprotect^a^ + isatuximab	1	Isatuximab
Daratumumab + lenalidomide	2	Lenalidomide
Daratumumab + ixazomib	1	Ixazomib
Ixazomib + lenalidomide	1	Lenalidomide
Selinexor + lenalidomide	1	Lenalidomide
Teclistamab + lenalidomide	1	Lenalidomide

^a^Cellprotect is a manufactured product consisting of invitro expanded and activated autologous NK cells

One study evaluating a regimen of ixazomib, lenalidomide, daratumumab and dexamethasone limited enrollment to standard risk disease (NCT03669445). No qualifying studies enrolled only “high-risk” disease. Among the 19 trials enrolling patients with high-risk disease, 8 (42.1%) permitted the use of single agent lenalidomide (Table 3).

**Table 3:** Clinical trials included in current analysis

Trial name	Trial identifier	Expected sample size	Randomized or non-randomized	Primary endpoint(s)
Belantamab mafodotin newly diagnosed transplant eligible multiple myeloma patients	NCT04802356	50	Non-randomized	Incidence of death, adverse events, analytical alterations, and ocular events
Study of belantamab mafodotin as pre- and post-autologous stem cell transplant and maintenance for multiple myeloma	NCT04680468	47	Non-randomized	MRD negativity rate
Iberdomide (Cc220) maintenance after asct in newly diagnosed MM patients	NCT04564703	160	Non-randomized	Efficacy, rate of dose reductions/discontinuations
Study association of lenalidomide, ixazomib, dexamethasone and daratumumab in newly diagnosed standard risk multiple myeloma	NCT03669445	45	Non-randomized	MRD negativity rate
Daratumumab after stem cell transplant in treating patients with multiple myeloma	NCT03346135	40	Non-randomized	PFS
Post autologous transplant maintenance with isatuximab and lenalidomide in minimal residual disease positive multiple myeloma (HEME-18)	NCT05344833	50	Non-randomized	MRD negativity rate
Belantamab mafodotin and lenalidomide for the treatment of multiple myeloma in patients with minimal residual disease positive after stem cell transplant	NCT04876248	20	Non-randomized	MRD negativity rate
A study of isatuximab added to standard cybord induction and lenalidomide maintenance treatments in ND-TEMM	NCT04786028	65	Non-randomized	Response rate (VGPR or better)
Iberdomide maintenance therapy in patients with multiple myeloma	NCT05177536	38	Non-randomized	Proportion of subjects who complete 1 year of therapy
Clinical trial for autologus nk cells alone or in combination with isatuximab as maintenance for multiple myeloma	NCT04558931	60	Randomized	Response rate (VGPR or better), change in MRD negativity rate
S1803, lenalidomide ± daratumumab/rHuPh20 as Post-ASCT maintenance for MM w/MRD to direct therapy duration (DRAMMATIC)	NCT04071457	1100	Randomized	OS
A study of daratumumab plus lenalidomide versus lenalidomide alone as maintenance treatment in participants with newly diagnosed multiple myeloma who are minimal residual disease positive after frontline autologous stem cell transplant (AURIGA)	NCT03901963	214	Randomized	MRD negativity rate
Daratumumab-bortezomib-dexamethasone (Dara-VCd) vs bortezomib-thalidomide-dexamethasone (VTd), then maintenance with ixazomib (IXA) or IXA-Dara	NCT03896737	400	Randomized	PFS, MRD negativity rate
An ALLG phase 3 randomized trial of selinexor and lenalidomide—versus lenalidomide maintenance post autologous stem cell transplant for patients with newly diagnosed multiple myeloma	ACTRN12620000291987	232	Randomized	PFS
A study of teclistamab in combination with lenalidomide versus lenalidomide alone in participants with newly diagnosed multiple myeloma as maintenance therapy following autologous stem cell transplantation (MajesTEC-4)	NCT05243797	1000	Randomized	PFS
Minimal residual disease guided maintenance therapy with belantamab mafodotin and lenalidomide after autologous hematopoietic cell transplantation in patients with newly diagnosed multiple myeloma	NCT05091372	94	Randomized	MRD positive to MRD negative conversion rate
A study of daratumumab, bortezomib, lenalidomide and dexamethasone (dvrd) followed by ciltacabtagene autoleucel versus daratumumab, bortezomib, lenalidomide and dexamethasone (DVRd) followed by autologous stem cell transplant (ASCT) in participants with newly diagnosed multiple myeloma (CARTITUDE-6)	NCT05257083	750	Randomized	PFS, sustained MRD negative CR
Isa-KRd vs KRd in newly diagnosed multiple myeloma patients eligible for autologous stem cell transplantation (IsKia TRIAL) (IsKia)	NCT04483739	300	Randomized	MRD negativity rate
Testing the addition of ixazomib to lenalidomide in patients with evidence of residual multiple myeloma, OPTIMUM Trial	NCT03941860	510	Randomized	OS, Change in FACT/GOG-Ntx TOI score, Change in FACT-MM TOI score
Study with elranatamab versus lenalidomide in patients with newly diagnosed multiple myeloma after transplant (MagnetisMM-7)	NCT05317416	366	Randomized	MRD negativity rate, PFS

In this review of currently enrolling MM maintenance trials, we demonstrate an encouraging variety of regimens being utilized, MRD-guided discontinuation approaches, as well as MRD-stratified enrollment being assessed. We note that OS is a primary endpoint in only a small minority of trials, and that many compare two drugs to lenalidomide alone, with an endpoint of PFS. Previous data from the FORTE trial have already shown that a two-drug combination (lenalidomide + carfilzomib) offers superior PFS to lenalidomide alone, raising questions as to whether there is true equipoise for the endpoint being studied in these randomized trials [10]. Furthermore, with the increasing number of active drugs available for patients with newly diagnosed MM, the question of optimal resource utilization arises. It may not be the best option to study all these drugs in a two versus one comparison in the maintenance setting against lenalidomide alone. A PFS endpoint is inherently biased towards combination therapy, which is currently being done for teclistamab (NCT05243797) and selinexor (ACTRN12620000291987).

It is taking increasingly longer to demonstrate an OS advantage in trials for patients with MM. Additionally, maintenance therapy is continuous, expensive, and may be toxic. Accordingly, the onus for maintenance therapy should be to change the natural course of the disease and not just delay disease progression [12]. In this setting, despite the logistical advantages, the use of PFS or MRD as an endpoint does not allow for ascertainment of whether a maintenance strategy is truly changing the course of the disease and helping patients live longer or better, or simply delaying time to biochemical progression. The use of PFS2, defined as time from initial randomization to disease progression on the "next-line" treatment or death from any cause, may serve as an acceptable alternative in this setting.

We note that quality of life (QOL) is seldom included in primary endpoint evaluations, although a study has suggested that a sizeable portion of the myeloma population prioritizes QOL metrics over PFS improvements [13, 14]. Implementation of QOL outcomes into these studies would allow for easier widespread adaptation of these therapies in a real-world population.

The current recommendation by USA guidelines for high-risk disease is doublet maintenance with lenalidomide and a proteasome inhibitors, although strong data beyond the FORTE trial for this recommendation are lacking [9]. Our study demonstrates that all currently enrolling randomized trials, except for one (NCT05091372), have control arms of single drug therapy, rendering high-risk patients potentially being subjected to treatment inferior to what is the prevailing standard of care. Future trials should address this, by designing specific risk-adapted strategies or specifically enrolling patients with high-risk disease. If delaying progression or deepening response is the goal of therapy, then the best potential standard of care should be offered to patients in the control arm, which may include doublet therapy.

Future trials should emphasize distinct maintenance strategies adapted for risk as well as depth of response. For example, a standard risk patient who has achieved a deep response (measurable residual disease negativity) following transplant, may be over-treated with intensive maintenance strategies incorporating two or three agents given for years. Indeed, for such patients, whether maintenance of any sort is necessary is a question worth revisiting. Conversely, a high-risk patient with residual disease may not be suited well on a lenalidomide control arm in these studies, and may be best included in clinical trials assessing novel combination maintenance approaches. As such, current “one size fits all” maintenance trials may indeed simultaneously over-treat and under-treat some patients.

Our current study has numerous limitations. While several databases and search terms were included in our search, some studies may have been missed. As we evaluated currently ongoing trials, we do not have access to the results of these.

This current study analyzing the landscape of current maintenance trials demonstrates that there are many promising designs that adapt treatment based on the depth of responses and incorporate newer agents. However, there remains room for improvement, most notably to identify the ideal maintenance regimens for patients with high-risk disease and inclusion of patient-centered endpoints.

## Data Availability

All data from which this study was generated is publicly available on clinical trials.gov.
